# Gene-Gene Interactions Lead to Higher Risk for Development of Type 2 Diabetes in an Ashkenazi Jewish Population

**DOI:** 10.1371/journal.pone.0009903

**Published:** 2010-03-26

**Authors:** Rosalind J. Neuman, Jon Wasson, Gil Atzmon, Julio Wainstein, Yair Yerushalmi, Joseph Cohen, Nir Barzilai, Ilana Blech, Benjamin Glaser, M. Alan Permutt

**Affiliations:** 1 Department of Psychiatry, Washington University School of Medicine, St. Louis, Missouri, United States of America; 2 Department of Genetics, Washington University School of Medicine, St. Louis, Missouri, United States of America; 3 Department of Internal Medicine, Washington University School of Medicine, St. Louis, Missouri, United States of America; 4 Institute of Aging, Department of Medicine and Genetics, Albert Einstein College of Medicine, New York, New York, United States of America; 5 Endocrinology and Metabolism Service, Hadassah-Hebrew University Medical Center, Jerusalem, Israel; 6 Diabetes Unit, The E. Wolfson Medical Center, Holon, Israel; 7 Endocrinology Unit, Clalit Health Organization, Tel-Aviv, Israel; 8 Diabetes Clinic, Macabbi Health Service, Tel Aviv, Israel; Uppsala University, Sweden

## Abstract

**Background:**

Evidence has accumulated that multiple genetic and environmental factors play important roles in determining susceptibility to type 2 diabetes (T2D). Although variants from candidate genes have become prime targets for genetic analysis, few studies have considered their interplay. Our goal was to evaluate interactions among SNPs within genes frequently identified as associated with T2D.

**Methods/Principal Findings:**

Logistic regression was used to study interactions among 4 SNPs, one each from HNF4A[rs1884613], TCF7L2[rs12255372], WFS1[rs10010131], and KCNJ11[rs5219] in a case-control Ashkenazi sample of 974 diabetic subjects and 896 controls. Nonparametric multifactor dimensionality reduction (MDR) and generalized MDR (GMDR) were used to confirm findings from the logistic regression analysis. HNF4A and WFS1 SNPs were associated with T2D in logistic regression analyses [P<0.0001, P<0.0002, respectively]. Interaction between these SNPs were also strong using parametric or nonparametric methods: the unadjusted odds of being affected with T2D was 3 times greater in subjects with the HNF4A and WFS1 risk alleles than those without either (95% CI = [1.7–5.3]; P≤0.0001). Although the univariate association between the TCF7L2 SNP and T2D was relatively modest [P = 0.02], when paired with the HNF4A SNP, the OR for subjects with risk alleles in both SNPs was 2.4 [95% CI = 1.7–3.4; P≤0.0001]. The KCNJ11 variant reached significance only when paired with either the HNF4A or WFSI SNPs: unadjusted ORs were 2.0 [95% CI = 1.4–2.8; P≤0.0001] and 2.3 [95% CI = 1.2-4.4; P≤0.0001], respectively. MDR and GMDR results were consistent with the parametric findings.

**Conclusions:**

These results provide evidence of strong independent associations between T2D and SNPs in HNF4A and WFS1 and their interaction in our Ashkenazi sample. We also observed an interaction in the nonparametric analysis between the HNF4A and KCNJ11 SNPs (P≤0.001), demonstrating that an independently non-significant variant may interact with another variant resulting in an increased disease risk.

## Introduction

Type 2 diabetes (T2D) has become one of the leading and fastest growing health problems in the world affecting approximately 18 million Americans and over 170 million individuals worldwide in 2000. By 2030 these estimates are expected to rise to over 30 million in the United States and 366 million worldwide [www.who.int/diabetes/facts/world_figures]. Although its exact etiology is unknown, accumulating evidence recognizes T2D as a quintessential multifactorial disease, resulting from numerous interactions between multiple genetic variants, along with environmental factors related to diet, exercise, stress, and medical treatment [1]. Given its position as one of the leading health problems in the world, in the last few years T2D has been the target of 15 genome wide association studies (GWAS) [2] and multiple candidate gene studies (reviewed in [3] and [4]). These studies have been successful in identifying individual variants in a variety of genes that may play a role in the etiology of T2D. However, because of practical and statistical challenges, none of the GWAS have considered interactions among the thousands of GWA variants, and only relatively few candidate gene studies have pursued such studies [5]–[13]. These studies generally include only those variants in their interaction analyses that have shown some nominal probability of marginal significance (e.g. p<0.05) in previous studies. However, there are genetic models in which variants show no independent association with the trait of interest, but which, in concert with other variants, do provide evidence of interaction effects [14]. This phenomenon has also been studied and documented in animal models [15], [16].

In the present study we investigated the importance of gene-gene interplay on type 2 diabetes in a case-control sample of Ashkenazi subjects. Four genes, TCF7L2, HNF4A, KCNJ11, and WFS1, all with strong prior evidence for association with T2D and with credible biological mechanistic effects on T2D, were chosen for analysis. One Single Nucleotide Polymorphism (SNP) was selected for genotyping from each of these four candidate genes based on previous studies in which the SNP was shown to be directly associated with T2D or in high linkage disequilibrium with a SNP identified as associated with T2D. SNPs in the TCF7L2 gene were selected as being the only truly universally T2D positive SNPs that had been identified at the start of this study. Two intronic SNPs in high LD (r^2^ = 0.77) in the TCF7L2 gene, rs12255372 and rs7903146, had been identified as being associated with T2D in at least 49 articles. We chose to genotype rs12255372 in our Ashkenazi population. HNF4A is a transcription factor regulating a network of genes controlling insulin secretion and glucose regulation. Recently we have found the HNF4A SNP, rs1884613, to be strongly associated with T2D in an Ashkenazi case-control sample unrelated to the sample in our current study [17]. Rs1884613 is located upstream of HNF4A and has no known function. The potassium channel gene, KCNJ11 on chromosome 11p15.1, regulates glucose-induced insulin secretion in pancreatic beta cells [18]–[20]. With the exception of Ashkenazi samples, coding SNP rs5219 (E23K) in KCNJ11 has been consistently associated with T2D populations over many years [21]. We included this SNP in our analysis to determine if its effect might be amplified by interactions with other genes in the Ashkenazi population. Although mutations in the WFS1 gene have long been known to be causal in autosomal recessive Wolfram syndrome [22], recently several studies have implicated WFS1 SNPs also to be associated with T2D [23]–[26]. Four of the five PubMed citations for T2D studies using rs10010131 found evidence for an association with T2D, including a meta analysis of previous studies from Sweden, Finland, and France, which reported a highly significant finding for this SNP [23]. In addition, rs10010131 was one of 2 intronic WFS1 SNPs in strong disequilibrium (r^2^ = 0.98) found to be significant in a study of T2D in UK and Ashkenazi populations studying genes involved in pancreatic β cell function [25] in both populations. Our multilocus analysis strategy makes use of the traditional parametric approach of logistic regression and the nonparametric method of multifactor dimensionality reduction (MDR) [27].

## Methods

### Ethics Statement

This study was approved by the local ethics committee of each center where samples were collected: the committee on Research Involving Human Subjects of The Hebrew University-Hadassah Medical School, Jerusalem, Israel; Committee on Clinical Investigations at Albert Einstein College of Medicine; the Human Research Protection Office at Washington University in St. Louis. Written informed consent was obtained from all participants before participation in the study.

### Samples

To minimize potential genetic heterogeneity, 1,870 subjects (974 cases, 896 controls) were recruited for a cross-sectional study from the Ashkenazi Jewish population. Blood was drawn from all subjects, and age of diagnosis was determined for cases by questionnaire and review of medical history. Subjects had been determined to have T2D if their fasting glucose was greater than 140 mg/dl on more than 1 occasion or random glucose was greater than 200 mg/dl on at least two occasions [28]. Ashkenazi Jews are a relatively homogeneous population thought to have developed from a founder population approximately 500 years ago [29]. Subjects who self identified as Ashkenazi were accepted into the study only if all four grandparents also self identified as Ashkenazi and were born in Northern or Eastern Europe. Most of the subjects in this study reside in Israel (N = 1,600); the remainder are United States citizens residing in New York City, ascertained from the Einstein Longevity Study [30], [31]. The NYC portion of our sample contained, with one exception, only controls. Sex, body mass index (BMI) and current smoking status were available for possible inclusion in the analyses.

DNA samples for the Israeli Ashkenazi subjects were obtained from Dr. Benjamin Glaser and genotyping data from the NYC sample were obtained from Dr. Gil Atzmon. Genotypes for Israeli Ashkenazim SNPs were assessed by PCR amplification of genomic DNA and Pyrosequencing technology (Biotage AB, Uppsala, Sweden) as previously described [32]. Genotypes for the NYC sample were assessed by Pyrosequencing technology (Biotage AB, Uppsala, Sweden) as discussed in [33].

### Data Analyses

Exact Hardy Weinberg Equilibrium (HWE) tests were performed for each SNP independently among cases and controls. Logistic regression models (SAS V9.1.3) were used to assess the effects of each of the 4 SNPs on T2D with and without adjusting for sex and body mass index (BMI). Smoking status was available for over 98% of controls, but only 25% of cases and therefore was not included in the data analyses. An additive model was used to code SNPs for the ‘risk’ allele, the allele that increased the probability of being a case. Accordingly, ‘0’ indicated the subject was homozygous for the non-risk allele; the heterozygote was coded ‘1’, and those who were homozygous for the risk allele were coded ‘2’. Interaction effects between SNPs were modeled using four mutually exclusive levels: (1) subjects without either risk allele, (2) subjects with one risk allele in the first SNP but without the risk allele in the second SNP, (3) subjects without the risk allele in the first SNP but with the risk allele in the second SNP, and (4) subjects with both risk alleles. Level (1), was considered the reference group [34], [35].

Multifactor dimensionality reduction (MDR, V2.0 Beta 2) was used to verify our interaction results. MDR was initially introduced by Ritchie et al., 2001, [36] as a genetically model-free and non-parametric alternative to logistic regression [27], [37]. A benefit of these non-parametric models is minimizing statistical issues that frequently arise when using traditional parametric models such as logistic regression. In particular, the possibility often exists that few or no observations will be assigned to contingency table cells when testing for interactions, thereby invalidating the resulting parameter estimates. Briefly, MDR acts by reducing a set of multilocus genotypes to one dimension with two groups: a high-risk and a low-risk set of genotypes. A particular multilocus genotype can be declared to be high-risk if the ratio of number of cases to controls exceeds the proportion of cases in the total sample. By grouping the high-risk multilocus genotypes together and the low-risk genotypes together, the model is reduced to one dimension, that is, essentially 1 variable with 2 possible values: high or low risk 2-locus genotypes. Models are evaluated on the testing balanced accuracy statistic (TBA) [38], the cross-validation consistency (CVC) [39], and the statistical significance of the model. The TBA measures how often individuals are correctly classified with respect to their case/control status, and the cross-validation consistency (CVC) evaluates the consistency with which individuals are classified. Heuristically, a satisfactory TBA score is above 0.55 (http://compgen.blogspot.com/2006/12/mdr-101-part-4-results.html). We used 10,000 permutations to determine the statistical significance of the best models. These data were also analyzed using an extension of the MDR algorithm that includes adjustment for covariates, the Generalized Multifactor Dimensionality Reduction (GMDR, V0.7) software package [40].

## Results

All SNPs were in Hardy-Weinberg equilibrium [P>0.23]. Characteristics of the cases and controls are displayed in Table 1. As expected, BMI was significantly greater among cases than controls [29.5 vs. 25.9, P≤0.0001]. Although there were significantly more males among cases than controls [49% vs. 42%, P = 0.002], the proportion among the controls recruited in Israel versus the U.S. did not differ [P = 0.82]. The age variable was highly correlated with the diagnosis of T2D; the mean age of examination for controls was 72 (±9.3) years versus 47 (±7.8) years for diagnosis of cases (p<0.0001). Accordingly, this variable was not included in the statistical analyses. The average age of cases when ascertained for this study was 64 years. Means and standard deviations for cases' HbA1c measurements and fasting glucose for controls are displayed in Table 1. HbA1c measures on controls were not recorded since at the time of ascertainment it was not an accepted diagnostic tool. Since glucose is totally controlled by treatment and can vary considerably from day to day, fasting glucose was not measured in the cases.

**Table 1:** Ashkenazi Sample Characteristics.

	Cases N = 974	Controls N = 896	Cases vs. Controls
Category	Mean ± STD	Mean ± STD	P-value
Age(1)	47.1±7.8	72.3±9.3	<0.0001
BMI	29.4±5.0	25.8± 4.2	<0.0001
Sex (% male)	49.5	42.2	0.0018
Smoking (% Current)	11.6	10.4	0.61
Fasting Glucose (mmol/L)(2)	NA	92.5±37.3	
HbA1c(2)	7.9±1.5	NA	
Site (% Israeli)	100.0	69.9	<0.0001

(1) Age at diagnosis for cases; age at examination for controls.

(2) Fasting Glucose was measured only in controls; HbA1c was measured only in cases.

As seen in Table 2, the genotype frequencies between cases and controls were significantly different for the HNF4A and WFS1 genotypes [P<0.0001, <0.0008, respectively]. We note that Ashkenazi cases had a greater excess of heterozygous and homozygous HNF4A genotypes with the risk allele than expected by chance, indicating a dominant mode of inheritance. Conversely, WFS1 case/control genotypes indicate a recessive mode of inheritance. Diverging from the results of multiple recent studies in non-Ashkenazi populations [41], the TCF7L2 genotype frequencies between cases and controls only minimally differed (P = 0.055), and the KCNJ11 genotypes showed no difference with the diagnostic status of the Ashkenazi subjects [P<0.55]. The OR and P-values of the logistic regression analyses on the additive genetic models for each SNP, with and without covariates, are displayed in Table 3. Of the four SNPs genotyped, only the HNF4A and WFS1 SNPs were significant with or without covariates after a Bonferroni correction for multiple tests; the P-value for rs12255372 in TCF7L2 was 0.02. Table 4 presents the results of models for the interaction effects of the genotypes. The risk of a diagnosis for T2D is greatest in subjects with the HNF4A and WFS1 SNP risk alleles versus those with neither risk allele [OR = 3.0, 95% CI = 1.7–5.3]. There is no or minimal increased risk for type 2 diabetes at these two loci if only one risk allele is present: OR = 1.8 [95% CI = 1.1–3.2] for the presence of the WFS1 risk allele but not the HNF4A risk allele, and OR = 1.8 [95% CI = 0.8–4.1] for the presence of the HNF4A risk allele but not the WFS1 risk allele. As noted in Table 3, the odds of T2D for subjects with the TCF7L2 risk allele was 1.2 times greater than subjects without the risk allele in the single SNP logistic regression model. However, when coupled with either the HNF4 or WFS1 genotype containing at least one risk allele, the unadjusted odds of being affected increased, respectively, to 2.4 and 2.9 times greater for subjects with the TCF7L2 risk allele than those with neither risk allele. There was no evidence for an increased risk for T2D due to the presence of the rs5219 [KCNJ11] risk allele interacting with any other risk alleles, with or without covariates.

**Table 2:** Case and Control Genotype Frequencies.

Gene (rs number)	Genotype	Cases (%)	Controls (%)
HNF4A (20q12-q13.1)	CC	276(52.5)	473(66.1)
(rs1884613)	CG	200(38.0)	215(30.1)
	GG	50(9.5)	27(3.8)
	Total	526(42.4)	715(57.6)
X^2^ 2df (P value)		31.2 (<0.0001)	
MAF		0.23	
KCNJ11 (11p15.1)	AA	79(13.8)	100(11.7)
(rs 5219)	AG	266(46.4)	404(47.9)
	GG	228(39.8)	339(40.2)
	Total	573(40.5)	843(59.5)
X^2^ 2df (P value)		1.18 (0.55)	
MAF		0.36	
TCF7L2 (10q25.3)	GG	217(37.7)	332(44.2)
(rs12255372)	GT	281(48.8)	334(44.4)
	TT	78(13.5)	86(11.4)
	Total	576(43.4)	752(56.6)
X^2^ 2df (P value)		5.82(0.055)	
MAF		0.35	
WFS1 (4p16,1)	CC	441(52.3)	318(44.6)
(rs10010131)	CT	343(40.7)	313(43.9)
	TT	59(7.0)	82(11.5)
	Total	843(54.2)	713(45.8)
X^2^ 2df (P value)		14.30 (0.0008)	
MAF(1)		0.30	

(1) The ‘risk’ allele for the WFS1 SNP is the major allele, C. The risk allele for all other SNPs is the minor allele.

**Table 3:** Associations between SNPs in candidate genes and T2D in the Ashkenazi population.

	OR[95% CI](1)	P-value	OR[95% CI](2)	P-value
HNF4A (rs1884613)	1.69[1.40–2.03]	<0.0001	1.77[1.39–2.24]	<0.0001
KCNJ11 (rs5219)	1.05[0.90–1.23]	0.52	1.02[0.83–1.26]	0.83
TCF7L2 (rs12255372)	1.21[1.03–1.42]	0.02	1.21[0.98–1.49]	0.07
WFS1 (rs10010131)	1.34[1.15–1.56]	<0.0002	1.30[1.06–1.58]	0.01

(1) Unadjusted.

(2) Adjusted by sex, BMI.

**Table 4:** Odds ratios and 95% Confidence Intervals (CIs) for the Joint Effects of Candidate SNP Genotypes on Type 2 Diabetes.

	Risk	Risk	Ashkenazi Sample	
Gene Pair	Allele 1(1)	Allele 2(1)	OR(95% CI)(2)	OR(95% CI)(3)
HNF4A, KCNJ11	−	−	1.00	1.00
(rs1884613,	−	+	1.21(0.88–1.66)	1.06(0.73–1.55)
rs 5219)	+	−	1.95(1.34–2.83)	2.25(1.40–3.62)
	+	+	1.96(1.39–2.77)	1.82(1.19–2.76)
HNF4A, TCF7L2	−	−	1.00	1.00
(rs1884613,	−	+	1.43(1.05–1.96)	1.33(0.91–1.94)
rs12255372)	+	−	1.88(1.29–2.74)	1.89(1.19–3.01)
	+	+	2.43(1.72–3.42)	2.47(1.61–3.78)
HNF4A, WFS1	−	−	1.00	1.00
(rs1884613,	−	+	1.82(1.05–3.17)	1.86(0.99–3.49)
rs10010131)	+	−	1.80(0.78–4.11)	2.46(0.91–6.63)
	+	+	2.99(1.70–5.27)	3.27(1.71–6.26)
KCNJ11,TCF7L2	−	−	1.00	1.00
(rs 5219,	−	+	1.24(0.87–1.76)	1.16(0.73–1.82)
rs12255372)	+	−	1.03(0.72–1.46)	0.89(0.57–1.39)
	+	+	1.34(0.97–1.85)	1.17(0.77–1.78)
KCNJ11,WFS1	−	−	1.00	1.00
(rs 5219,	−	+	2.32(1.21–4.46)	2.70(1.30–5.60)
rs10010131)	+	−	1.71(0.74–3.98)	2.04(0.78–5.31)
	+	+	2.32(1.22–4.41)	2.17(1.07–4.44)
TCF7L2,WFS1	−	−	1.00	1.00
(rs12255372,	−	+	2.18(0.97–4.90)	2.18(0.87–5.45)
rs10010131)	+	−	1.60(0.62–4.09)	1.68(0.58–4.85)
	+	+	2.87(1.28–6.40)	2.69(1.09–6.66)

(1) Risk alleles 1 and 2 refer to the leftmost and rightmost genes in the ‘Gene Pair’column. ‘−’ indicates absence of the risk allele; ‘+’ indicates presence of the risk allele; allele1 and allele2 = ‘−’ is the reference group.

(2) Unadjusted.

(3) Adjusted for Sex, BMI.

Results from the multilocus MDR and GMDR analyses are summarized in Table 5. The CVCs indicate consistency in the cross validation measures (10/10) across all models. Based on the testing balanced accuracy (TBA) statistic and permutation p-values, the most significant interactions, adjusted and unadjusted for covariates, were pairs containing the HNF4A SNP: HNF4A x KCNJ11 (P = 0.001), HNF4A x TCF7L2 (P = 0.03), and HNF4A x WFS1 (P = 0.009). Adding covariates strengthened the results for HNF4A x TCF7L2 (P<0.002) and HNF4A x WFS1 (P<0.004) interactions. TCF7L2 x WFS1 (P = 0.01) was also significant, but not with the inclusion of covariates. Over all, the parametric and nonparametric results were consistent for the significant interactions between HNF4A x KCNJ11, HNF4A x TCF7L2, and HNF4A x WFS1. Consistency between the logistic regression and MDR methods was also observed in the KCNJ11 x TCF7L2 analyses in which neither method indicated a significant interaction between the SNPs in these genes. The KCNJ11 x WFS1 interaction was not significant in the MDR analyses nor in the logistic regression if no WFS1 risk allele was present. Higher order three way unadjusted MDR interactions were nominally significant only if the HNF4A SNP was one of the interacting SNPs. Among the three-way unadjusted interactions, HNF4A x KCNJ11 x TCF7L2 was the most significant interaction: P≤0.001 (Table 5). To compare the parametric and non-parametric methods, we analyzed the same three SNPs using logistic regression, albeit, in contrast to an MDR or GMDR analysis, contingency table cells that contain few or no observations would result in unstable results, a situation that may occur when interactions are introduced into the model [42]. A significant association with T2D was found: OR = 2.4, CI = [1.5–4.0], P≤0.0006. We also note that the MDR interaction between all four SNPs was not significant without inclusion of sex and BMI. But that increase was extremely modest (P = 0.03).

**Table 5:** Gene x Gene Interaction Models: MDR (without covariates) and GMDR (with covariates).

Interacting SNPs	Covariates	Number of Subjects	CVC(1)	TBA(2)	10,000 Permutations P-value
HNF4A x KCNJ11	none	1162	10/10	0.560	0.001
	Sex,BMI	853	10/10	0.564	0.002
HNF4A x TCF7L2	none	1153	10/10	0.540	0.028
	Sex,BMI	848	10/10	0.565	0.002
HNF4A x WFS1	none	1176	10/10	0.546	0.009
	Sex,BMI	829	10/10	0.559	0.004
KCNJ11 x TCF7L2	none	1288	10/10	0.504	0.437
	Sex,BMI	918	10/10	0.495	0.595
KCNJ11 x WFS1	none	1117	10/10	0.516	0.252
	Sex,BMI	813	10/10	0.528	0.146
TCF7L2 x WFS1	none	1111	10/10	0.546	0.010
	Sex,BMI	812	10/10	0.535	0.090
HNF4A x KCNJ11 x TCF7L2	none	1122	10/10	0.565	0.001
	Sex,BMI	836	10/10	0.546	0.040
HNF4A x KCNJ11 x WFS1	none	1105	10/10	0.541	0.031
	Sex,B MI	802	10/10	0.549	0.034
HNF4A x TCF7L2 x WFS1	none	1096	10/10	0.551	0.011
	Sex,BMI	798	10/10	0.540	0.072
KCNJ11 xTCF7L2 x WFS1	none	1080	10/10	0.528	0.122
	Sex,BMI	799	10/10	0.497	0.549
HNF4A x KCNJ11 x TCF7L2 x WFS1	none	1068	10/10	0.512	0.313
	Sex,BMI	788	10/10	0.552	0.029

(1) Cross Validation Consistency.

(2) Testing Balanced Accuracy.

## Discussion

Our main finding is that genetic interactions modulate the risk for T2D in the Ashkenazi case-control sample. Specifically, the strength of the association between polymorphisms of the HNF4A and WFS1 genes and T2D in the parametric analyses indicated a three fold increased risk of T2D for individuals who carry the risk alleles of these polymorphisms compared to individuals whose genotypes contain neither risk allele. This interaction was also echoed in the MDR/GMDR analysis. Although the TCF7L2 SNP was not strongly associated with T2D in this population, when TCF7L2 was jointly analyzed with either of the HNF4A or WFS1 SNPs, more than a two-fold increase in T2D risk was observed for subjects with both risk alleles. This was also borne out in the nonparametric MDR/GMDR analyses. While this phenomena has been discussed in a theoretical context [14] and has been shown to exist in animal models [15], [16], it has not commonly been seen in human studies, particularly one with thousands of variants, such as a GWA study in which any SNPs that are not highly significant are often not included in follow-up interaction or non-interaction studies. In this analysis there was no evidence that the KCNJ11 SNP, rs5219, altered the strength of the association in the parametric analysis when paired with any other candidate SNP. The apparent importance of rs5219 in the nonparametric interaction analysis may have been the result of the presence of rs1884613 [HNF4A] in the interaction models. However, it is difficult to understand the disparity in the significance between the nonparametric HNF4A x KCNJ11 x TCF7L2 interaction (P≤0.001), and the HNF4A x TCF7L2 x WFS1 interaction (P≤0.01) given the significance of the WFS1 versus the KCNJ11 SNPs in the logistic regression analyses. To the best of our knowledge, this is the first time a significant genetic interaction effect associated with T2D was reported in a sample of Ashkenazi Jewish descent.

Among those who have reported on studies involving variants in genes of interest in the current study is Weeden et al., 2006 [13] who examined the relationship among rs5219 in the KCNJ11 gene, rs7903146 in TCF7L2, a SNP in high LD with rs12255372 (r^2^ = 0.75), and Pro12 in the PPARG gene in a sample of over 6,000 subjects from the UK. Although no gene-gene interactions were found, a strong positive relationship between the numbers of risk alleles within a subject and risk of T2D was reported: subjects with all six risk alleles had an OR of 5.7 [95% CI = 1.2–28.3] for T2D compared to subjects with no risk alleles. Cauchi et al., 2008 [6] chose 22 SNPs in 14 loci from a previous GWA study to replicate in four independent European samples, including an Israeli Ashkenazi and Moroccan sample. However, they only analyzed interaction effects in the French subset of their sample, and none of their loci overlapped with any of those analyze in the current study. Using MDR, Qi et al. [9] reported an interaction between rs5219 in KCNJ11 and rs2144908 in HNF4A in a case-control study of T2D in a sample of female subjects from the Nurses' Health Study. We note that rs2144908 is in complete LD with rs1884613, r^2^ = 1. Therefore, our MDR finding of an interaction between rs5219 and rs1884613 in the Ashkenazi sample is a replication of the Qi et al. study. To the best of our knowledge, Qui et al. was the first to report an interaction between SNPs in the HNF4A and KCNJ11 genes.

A strength of the current study is that all subjects with T2D were Ashkenazi Jews living in Israel. Almost 70% of the controls were also ascertained in Israel, with the remainder Ashkenazi Jews from the U.S. No differences were detected between the two groups of controls regarding smoking status, BMI, or gender. By choosing Ashkenazi Jews for both cases and controls, we have minimized the potential of genetic heterogeneity. An additional strength of this study is that the mean age at ascertainment of non-T2D status in the control population was 76 years, whereas the mean age of T2D diagnosis in the cases was 25 years less, adding to the confidence that this control group is not at high risk for a future diagnosis. Furthermore, the average age at diagnosis of the cases was 47 years, relatively young, and therefore more likely to be genetically predisposed to T2D [43].

A major challenge for studies such as ours and those that investigate the interaction between thousands of variants is to decipher the complexity of the genetic networks between the loci that appear to have a statistical relationship [44]. In this study, we know that WFS1 appears to be part of the mechanisms related to beta-cell survival [45], and HNF4a may have proliferative and/or functional effects. The functional effects may interact with survival–increased function resulting in increased endoplasmic reticulum (ER) and/or oxidative stress resulting in increased apoptosis. Inactivating mutations in HNF4a have been shown to cause hyperinsulinemic hypoglycemia during the fetal and newborn period, as well as MODY diabetes in adolescence [46]. We still know very little about whether T2D associated variants effects increased or decreased expression of HNF4A. The mechanism for such effect is still not well understood. Therefore it would be pure speculation to try to explain the interactions found in this study on the basis of known function.

In conclusion, all four chromosomal regions investigated in this study harbor T2D genetic variants that have been replicated in published studies, but very few studies have considered their interactions. Nevertheless, we fully expect that additional variants, some rare, will be added to the growing list of polymorphisms that influence T2D, and that the analyses of interacting variants will lead to a better understanding of the biological pathways underlying T2D. This knowledge will bring us closer to the development of effective prevention and treatment strategies.
